# Is Vaginal Hysterectomy Safe for an Enlarged Uterus?

**DOI:** 10.7759/cureus.6816

**Published:** 2020-01-30

**Authors:** Pinar Yalcin Bahat, Sibel Gülova, Bahar Yuksel Ozgor, Kubra Cakmak

**Affiliations:** 1 Obstetrics and Gynecology, Kanuni Sultan Süleyman Training and Research Hospital, Istanbul, TUR; 2 Obstetrics and Gynecology, Sisli Hamidiye Etfal Training and Research Hospital, Istanbul, TUR; 3 Obstetrics and Gynecology, Esenler Maternity and Children’s Hospital, Istanbul, TUR; 4 Obstetrics and Gynecology, Esenler Maternity and Children's Hospital, Istanbul, TUR

**Keywords:** vaginal hysterectomy, enlarged uterus, uterine size

## Abstract

Objective

The purpose of this study was to compare the surgical outcomes between two sets of women undergoing vaginal hysterectomy (VH) for benign gynecological conditions: those with moderately enlarged ( ≥12 weeks') uteruses and those with normal-sized uteruses.

Materials and Methods

The medical records of 84 women who underwent vaginal hysterectomies for benign gynecological conditions at Şişli Hamidiye Etfal Training and Research Hospital, Istanbul, Turkey between 2013 and 2015 were reviewed. Age, uterine sizes, indications, duration of hospitalization, operation time, hematocrit (HCT) levels, and complications were analyzed.

Results

The most common indications for VH were uterine descensus. However, most women had presented with more than one indication. The mean age of the patients who underwent VH was 56.12. The maximum volume of the uterus was found to be 1244.74 ml, and the smallest volume was found to be 18.83 ml. The mean volume of the uterus was found as 122.6629 ml. The mean duration of operation was 159.70 minutes, whereas the mean duration of hospital stay was 3.79 days. The mean preoperative HCT and hemoglobin (Hgb) values were 37.098 (±3.64) gr/dl and 12.365 (±1.35) gr/dl respectively. Postoperative HCT and Hgb values were 31.363 (±3.94) gr/dl and 10.52 (±1.38) respectively.

Conclusion

VH is usually a simple procedure with low morbidity. It is important to choose the appropriate patient when deciding on the operation. In addition, having experienced surgeons in the field of VH increases the success of surgery.

## Introduction

Hysterectomy, performed for various indications, is the most frequent among major gynecological surgeries [1]. There are three types of hysterectomy procedures: abdominal, vaginal, and laparoscopic. Vaginal hysterectomy (VH) has advantages in aesthetics and recovery time compared to the abdominal approach [2]. However, VH feasibility is limited by uterine size and concomitant lesions within the abdominal cavity. Also, the range of indications for VH may vary greatly depending on the level of experience of the surgeon [3]. When VH is not indicated, abdominal hysterectomy is preferred; yet physicians may have different approaches to the same clinical circumstances, depending on training and background [4-6]. The purpose of this study is to analyze the characteristics of patients who have undergone VH.

## Materials and methods

A retrospective chart review was performed for 84 patients who underwent VH between 2013 and 2015 at Şişli Hamidiye Etfal Training and Research Hospital, Istanbul, Turkey. Age, uterine size, indication, duration of hospitalization, operation time, hematocrit (HCT) level, and complications were analyzed. Exclusion criteria were a uterine size larger than 10 gestational weeks' size and one or more of the following conditions: prior pelvic surgery, a history of the pelvic inflammatory disease, moderate or severe endometriosis, accompanying adnexal masses, indication for adnexectomy, and nulliparity without uterine descent. All patients except those with a penicillin allergy received prophylactic antibiotic treatment (cefazolin 2 g, intravenous) at the beginning of the operation. All patients received prophylactic anticoagulant therapy with low molecular weight heparin 12 hours after the operation. All operations were performed with patients under general anesthesia.

In this retrospective cohort study, IBM SPSS Statistics for Windows, Version 23.0 (IBM, Armonk, NY) was used for statistical analysis. The suitability of the quantitative data for normal distribution, according to uterine groups, was examined by the Kolmogorov-Smirnov test. Since the data did not fit a normal distribution, the comparisons were examined by the Mann-Whitney U test. The Wilcoxon test was used for pre- and post-comparison of hemoglobin (Hgb) and HCT values for the groups. Categorical data were analyzed by the chi-square test. A p-value of <.05 was considered statistically significant.

Uterine volume was calculated using the ellipsoid formula, as follows: longitudinal length (cm) x transverse length (cm) x anteroposterior length (cm) x 0.523 ( the number 0.523 corresponds to Π/6) [7,8]. Group 1 was defined as those having a uterine volume of ≤98 ml while those with a volume of >98 ml were placed in Group 2.

## Results

Fifty patients had a uterine volume of ≤98 ml, and 34 patients had uterine volume >98 ml. The indications for VH are listed in Table 1. The most common indication for VH was uterine descensus. However, most of the women presented with more than one indication. The demographic data of the patients are listed in Table 2. The mean age of the patients who underwent VH was 55 years (range: 40-80 years). Prophylactic bilateral salpingo-oophorectomy was performed for 43 patients. Anterior colporrhaphy was also done for 44 patients; posterior colporrhaphy was added to the operations of 39 patients, and transobturator tape was additionally added for seven patients, while sacrospinous fixation was performed on 23 patients receiving VH. The mean uterine volume was 75.1 ml (range: 18.8-1244.7 ml).

**Table 1:** Preoperative diagnosis *Most patients presented with more than one indication **Total number of patients: 84

Diagnosis*	Number of patients** (%)
Uterine descensus	52 (61)
Leiomyoma	19 (22)
Cystocele	20 (23)
Rectocele	8 (9)
Urinary incontinence	12 (14)
Dysfunctional uterine bleeding	3 (3)

**Table 2:** Comparison of quantitative data by groups *Figures in parentheses represent the range **Mann-Whitney U test HCT: hematocrit; Hgb: hemoglobin

Characteristics, mean	Group 1, n = 50	Group 2, n = 34	Total, n = 84	P-value**
Age, years	59.5 (40-80)*	50 (41-70)*	55 (40-80)*	.000
Gravida, n	6 (1-14)*	4 (1-10)*	5 (1-14)*	.015
Parity, n	4 (1-13)*	3 (1-9)*	3.5 (1-13)*	.016
Abortion, n	0 (0-5)*	0 (0-7)*	0 (0-7)*	.467
Curettage, n	0 (0-6)*	0 (0-5)*	0 (0-6)*	.300
Operation duration, minutes	150 (50-300)*	175 (60-300)*	150 (50-300)*	.291
Hospitalization time, days	3.5 (1-12)*	4 (2-8)*	4 (1-12)*	.259
Longitudinal length, cm	8 (4.5-12)*	10 (7.5-17)*	9 (4.5-17)*	.000
Transverse length, cm	4 (3-6.5)*	6 (5-14)*	5 (3-14)*	.000
Anteroposterior length, cm	2.5 (1.5-5)*	5 (4-10)*	3.5 (1.5-10)*	.000
Volume, ml	47.3 (18.8-95.4)*	162.4 (100.4-1244.7)*	75.1 (18.8-1244.7)*	.000
Preoperative Hgb, g/dl	12.7 (9.2-15.3)	12.2 (5.8-14.2)*	12.4 (5.8-15.3)*	.016
Preoperative HCT, g/dl	37.9 (29.4-44)*	36.2 (18.8-42.4)*	37.6 (18.8-44)*	.079
Postoperative Hgb, g/dl	11 (6-13.7)*	10.6 (7.8-12.4)*	10.7 (6-13.7)*	.066
Postoperative HCT, g/dl	32.4 (19.2-40.1)*	31.5 (23.9-37.2)*	32 (19.2-40.1)*	.125

The mean preoperative HCT and Hgb values were 37.6 g/dl (range: 18.8-44 g/dl) and 12.4 g/dl (range: 5.8-15.3 g/dl), respectively; whereas postoperative HCT and Hgb values were 32 g/dl (19.2-40.1 g/dl) and 10.7 g/dl (range: 6-13.7 g/dl), respectively. There was no significant difference between the two groups in terms of changes in uterine volume or HCT and Hgb values (Hgb p: .066; hct p: .125). The mean duration of the operations was 150 minutes (range: 50-300 minutes), whereas the mean hospitalization time was four days (range: 1-12 days).

Menopausal status varied between the groups (p: <.001); 88% of patients in the small uterus and 23.5% in the large uterus group were menopausal. Data pertaining to smoking, diabetes mellitus, hypertension, coronary artery disease, thyroid conditions, previous abdominal surgery history, transfusion, and complications were not much different between the groups (p: >.05; Table 3).

**Table 3:** Comparison of categorical data by groups *Chi-square test

Characteristics, mean	Group 1, n (%), total number = 50	Group 2, n (%), total number = 34	Total n (%), total number = 84	P-value*
Menopause				
No	6 (12)	26 (76.5)	32 (38.1)	.001
Yes	44 (88)	8 (23.5)	52 (61.9)	
Cigarette use				
No	44 (88)	30 (88.2)	74 (88.1)	1.000
Yes	6 (12)	4 (11.8)	10 (11.9)	
Diabetes mellitus				
No	44 (88)	32 (94.1)	76 (90.5)	.464
Yes	6 (12)	2 (5.9)	8 (9.5)	
Hypertension				
No	32 (64)	23 (67.6)	55 (65.5)	.911
Yes	18 (36)	11 (32.4)	29 (34.5)	
Coronary artery disease				
No	48 (96)	29 (85.3)	77 (91.7)	.113
Yes	2 (4)	5 (14.7)	7 (8.3)	
Thyroid disease				
No	41 (82)	26 (76.5)	67 (79.8)	.732
Yes	9 (18)	8 (23.5)	17 (20.2)	
Previous abdominal surgery				
No	34 (68)	29 (85.3)	63 (75)	.124
Yes	16 (32)	5 (14.7)	21 (25)	
Transfusion				
No	46 (92)	30 (88.2)	76 (90.5)	.709
Yes	4 (8)	4 (11.8)	8 (9.5)	
Complication				
No	48 (96)	34 (100)	82 (97.6)	.512
Yes	2 (4)	0 (0)	2 (2.4)	

Five women required blood transfusion postoperatively because of excessive hemorrhage. Four women needed blood transfusions before their operations because of anemia. Bladder injury occurred in two women after VH. The injury was noticed during the operation and repaired perioperatively. There were no ureter or bowel injuries, vesicovaginal fistulae, neurologic or thromboembolic complications identified during the study. Four patients underwent laparotomy due to unstoppable excessive bleeding during VH. Patients with a uterine volume of ≤98 ml and those with a uterine volume of >98 ml were both evaluated for complications and transfusion. There was no difference in complication and transfusion rates between the groups (complication: p: .512; transfusion: p: .709).

## Discussion

In obstetrics and gynecology, hysterectomy is the second most commonly performed surgery after the Cesarean section [9]. The patient's anatomy and the surgeon's experience are highly important for choosing the type of hysterectomy [10]. Despite the advantages of the VH, most surgeons hesitate to perform the procedure when faced with a large uterus, previous history of pelvic or salpingo-oophorectomy surgery, pelvic inflammatory disease, severe endometriosis, adnexal mass, or descensus in non-uterine cases.

Feroze et al. reported indications for VH as benign uterine diseases, a size not greater than which corresponding to a 12-gestational week uterus (<280 g), uterine prolapse, small leiomyomas, cases with severe dysmenorrhea, and cases with functional uterine bleeding concomitant with uterine prolapse [10]. Subsequent studies have led to some changes in terms of indications for VH. For example, Kammerer-Doak et al. and Mazdisnian et al. reported no prominent distinction according to the size of the uterus [11,12]. Although the indications have been expanded, there are still some limitations for VH, such as prior pelvic surgery, a history of pelvic inflammatory disease, moderate or severe endometriosis, accompanying adnexal masses, indication for adnexectomy, and nulliparity without uterine descent [9].

In a study conducted by Amy in 1997, VH was performed for 14 cases with uterine weight up to 639 g, and they found no difference in terms of complications [13]. As Magos et al. concluded, uterine size by itself should no longer be considered as a contraindication for VH. As reported previously, prior pelvic surgery, mild endometriosis, history of pelvic inflammatory disease, or leiomyoma should no longer be viewed as contraindications for vaginal surgery [14].

In our study, 20 patients had a history of Cesarean section, and six patients had undergone prior surgery due to various indications. However, no complications occurred. In our study, the most common indication for VH was descensus uteri, which is consistent with the literature [15,16].

Prophylactic adnexectomy is much less frequent in VH due to technical issues. In a study by Wilcox et al., prophylactic oophorectomy was performed in 85% of the women who had abdominal hysterectomies, while only 18% of women had oophorectomy with VH [17]. In our study, 43 patients had prophylactic bilateral salpingo-oophorectomy.

In a study published in the US, the overall complication rate was 42.8% in abdominal hysterectomies and 24.5% in VH. The risk of developing one or more complications after surgery is 1.7-times greater in abdominal hysterectomies [18]. In our study, bladder injuries occurred in two (2%) patients during vaginal hysterectomy and four (4%) patients during laparotomy due to excessive bleeding. Only five patients needed blood transfusion postoperatively due to blood loss.

No correlation was found between uterine volume and operation time or discharge time: 50 of the 84 patients (59.5%) included in the study were in the small uterine group, and 34 (40.5%) were in the large uterine group.

## Conclusions

Although VH has obvious advantages compared with other techniques, a detailed examination should be performed as part of the treatment planning process. If there is a contraindication, one should not insist on it; rather, one should switch to an abdominal or laparoscopic technique. Consequently, the success rate of the operation may increase with the presence of correct indications and the availability of a sufficiently experienced surgeon.
